# Handy numerals: compositional elements in body-based numeration systems

**DOI:** 10.1098/rstb.2024.0215

**Published:** 2025-10-20

**Authors:** Olga Dudojć, Andrea Bender, Chiara Anceschi

**Affiliations:** ^1^ Department of Psychosocial Science, University of Bergen, Bergen, Norway; ^2^ Centre for Early Sapiens Behaviour (SapienCE), Department of Archeology, History, Cultural Studies and Religion, University of Bergen, Bergen, Norway

**Keywords:** finger counting, numeration systems, compositional element, number representation, numeral base, embodied cognition

## Abstract

Body-based numeration systems represent numbers through the use of body parts and hence are subject to constraints imposed by human anatomy. Body parts are readily available and can be manipulated in three-dimensional space, but are also predetermined in shape and quantity, which creates both opportunities and obstacles. Strategies to produce composite numerals include the recruitment of compositional elements, such as numeral bases. This article aims to uncover which types of compositional elements (‘compositional anchors’) are present in body-based numeration systems and how they are represented. Analysing the data from the *BodyBase* database, we find that compositional elements appear in various types and values, showing both similarities with and differences from verbal and notational systems. The representations of main compositional anchors employ various strategies, including different types of movements, hand shapes and physical space. We discuss patterns of compositionality in body-based systems and how they are shaped by format-specific characteristics, and compare the systems of body-based representations with systems in other formats.

This article is part of the theme issue ‘A solid base for scaling up: the structure of numeration systems’.

## 1. Introduction

The human body facilitates the expression, retention and manipulation of numerical information. Fingers and other body parts provide practical means to enumerate objects and visually convey numbers to others (known as 'montring' [1]), perform arithmetic operations [2,3] and implement conventionalized numeral systems in sign languages (e.g. [4]). Despite the physical constraints imposed by human anatomy, such as the fixed number of anatomical parts and their locations, or the limitations of gesture production [5,6], body-based systems offer a wide range of methods for numerical representation, and they differ in ways that go far beyond the usage of two collections of five extended fingers to count to 10.^1^ Practices vary in terms of the body parts recruited for counting and the strategies employed to expand the numeral sequence [7–9]. Even below 10, the representation of numbers can vary significantly, for instance in whether and how fingers are extended, flexed, grasped or manipulated in other ways. Crucially, this variability reveals a remarkable resourcefulness in incorporating bases and other compositional elements used to recursively compose larger expressions from a limited set of numeral symbols (for the representation of internal structure of body-based numeration systems, see [10]). Dynamic gestures, symmetry of hands and relative flexibility of fingers are just some of the features used to introduce compositional elements, which in turn increase the structural compactness and productivity of the system.

Theoretical discussions of numeral bases and compositional elements have focused predominantly on notational systems [11–14] and verbal systems (e.g. [15–18]), leaving other representational formats underexplored [7]. The present article addresses this gap in knowledge by focussing on body-based numeration systems and their compositional elements. After clarifying key concepts and terminology, and describing the data collection, we provide an overview of the different types of compositional elements and their numerical values, and outline how bases and other main compositional elements are represented in these systems. Following this, we discuss emerging patterns, including format-specific characteristics affecting compositional elements and similarities, differences and interactions with other representational formats.

## 2. Key concepts and terminology for analysis

This paper focusses on body-based representations of numbers, aiming to do so in a manner that will ultimately allow for comparison with numeration systems in other representational formats, such as verbal and notational systems [11,19–21]. To achieve this, it is crucial to adopt a shared theoretical framework across formats. When explaining the key concepts used in the analysis of the dataset presented in this paper (§§3 and 4), we therefore follow the recommendations for conceptual and terminological coherence advocated by Pelland [22,23] and Barlow [21].

### (a) Types of compositional elements

Compositional elements are the building blocks of non-atomic numerals. *Atoms* are the smallest meaningful units; they cannot be broken down into smaller parts representing numbers, but can be combined through arithmetic operations (such as addition, multiplication, subtraction or exponentiation) in order to compose non-atomic numerals. The number to which such an operation is applied is termed the *compositional anchor* [23], with the most frequently used anchor being the *main anchor* of the system. A *numeral base* is a special case of main anchor, in that it also requires a distinct representation of at least two non-zero powers of that number marked within the system [23].

Sometimes, bases and main anchors operate alongside other anchors, such as sub-bases, sub-anchors, secondary anchors and sporadic anchors [22]. A *sub-base* can appear in systems containing a base, where it then initiates additional shifts in the counting unit (for instance, think of the Roman notational system, where the sub-base 5 [V] is used to compose numerals such as ‘VII’ and reoccurs in higher units such as the numerals for 50 [L] and 500 [D]). A *sub-anchor* is analogous to a sub-base, yet in those systems whose main anchor is not a base. A *secondary anchor* has functions similar to a main anchor, in that it helps to structure the system, but it does so less frequently and only in limited ranges (an example can be found in French, which for most parts is decimal but shifts to compositions including 20 [*vingt*] as secondary anchor in the words from 80 [*quatre-vingts*, literally ‘four twenties’)] to 99 [*quatre-vingt-dix-neuf*, literally ‘four twenties ten nine’]). A *sporadic anchor*, finally, is used irregularly to compose non-atomic numerals (an illustration of this is the Russian word for 40 (*sorok*), which deviates structurally from the composition of other tens and functions as an anchor for nine other numerals, 41−49, only).

As fingers naturally occur in fixed quantity and position, numerals based on them often stand in one-to-one correspondence with the number they represent. In previous accounts, European finger-counting systems, where fingers are extended sequentially on both hands until reaching 10, have often been categorized as base−10 (and occasionally sub-base−5) systems (e.g. [24,25]). However, we advocate that merely grouping five fingers on one hand or transitioning from one hand to another *does not*, in itself, constitute a base (and sub-base) system [7,26]. Maintaining a stricter concept of base is also warranted by its actual existence in body-based systems around the world, and by the creativity these systems demonstrate in using such bases to form more complex numerals.

Identifying the type of anchor in body-based systems poses several challenges, beginning with determining the atomicity of numerals and shifts in counting units. For illustration, take a system in which fingers are extended one by one to signify numbers from 1 to 4, followed by a fist to represent 5. Here, the numeral for 5 is atomic because it cannot be dissected into constituent parts with a numerical value of their own. Although the fist is formed from five bent fingers, bent fingers are not systematically used to represent numerals below 5—bending two fingers to signify 2 does not convey the intended meaning and is not a conventional practice.

The challenge in identifying atomic numerals is compounded further when symbol formation involves nuanced changes. Consider the case just described, but now with the numeral for 4 represented by a gesture similar to the ‘Vulcan salute’ in Star Trek (i.e. by grouping index with middle finger, and ring finger with pinkie, separated by a V-shaped gap, with the thumb remaining close to the palm). The formation rules producing this numeral read as (i) ‘raise the appropriate number of fingers’ and (ii) ‘group them together symmetrically’, but the numerals for 1–3 follow rule (i) only. Moreover, the numeral for 4 cannot be decomposed into lower meaningful numerals: neither the numeral for 2 nor the numeral for 3 is distinctly visible within the numeral for 4, as they do not involve the grouping of fingers. Thus, a numeral is categorized as atomic if its formation rules are unique.

The final challenge arises from the versatility of human hands, which renders movements conveying meaning a distinctive feature compared to other formats. As independent semantic units, movements can alter the numerical value of accompanying handshapes [27]. For instance, a closed fist representing 5 can be paired with a shaking movement (signifying 'times 10') to represent 50 (this aspect is illustrated in the example below and further explored in §4c(ii)).

Table 1 illustrates some of the building blocks described above with the Pokot (previously referred to as Suk) numeration system [28]. This system uses two anchors: while 5 is the system’s *smallest* anchor (used to compose the numerals for 6–9), its *main* anchor is 10, because this is the most frequently used number for composing non-atomic numerals; and 10 also qualifies as a *base,* because its next power is likewise distinctively represented. This renders 5 a *sub-base*, because it shifts the counting unit both below the base and above it.

**Table 1.** The Pokot body-based numeration system transcribed into gestures for numbers, rules for forming those numerals and the composition of numerical content. Sub-base and base are shown in bold.

no.		gesture description	formation rules		composition
1	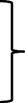	1−3 fingers extended	A (= ‘extend’)	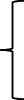	1
2					1+1
3					1+1+1
4		‘Vulcan salute’	A+B (= ‘group’)		4
**5**		fist	C (= ‘fist’)		**5**
6	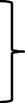	1−4 fingers of one hand inserted in the fist of another hand	C+A	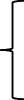	**5**+1
7					**5**+1+1
8					**5**+1+1+1
9					**5**+1+1+1+1
**10**		flick of index finger	D (= ‘new gesture’)		**10**
11	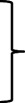	1−4 fingers bent	E (= ‘bend’)	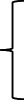	+1’
12					+1’+1’
13					+1’+1’+1’
14					+1’+1’+1’+1’
15		10-sign followed by 5-sign	D+C		**10+5**
16		10-sign followed by 5- and 1-sign	D+C+A		**10+5**+1
…					
20		fingers flapped against palm	F (= ‘new gesture’)		20
21		20-sign followed by 1-sign	F+A		20+1
…					
30		thumb and middle joined, index extended	G (= ‘new gesture’)		30
40		4-sign, hand shaken	AB+H (= ‘motion’)		4 ∙ **10**
50		5-sign, hand shaken	C+H		5 ∙ **10**
100		flick of all fingers	I (= ‘new gesture’)		100

### (b) Representation of anchors

While the *type* and *value* of anchors (including full bases) are, first and foremost, pivotal for the structure of a system, the way in which anchors are *represented* is directly and critically relevant for cognitive processing. Specifically, which types of numerical information are represented in an implicit versus explicit manner generates a *representational effect*, insofar as it affects working memory, number processing, and task difficulty [12,14,16,29]. Base representation is covered in all major typologies (e.g. [11,13,14]), albeit with slight variations in terms of how representational principles for multipliers, bases, and sub-bases are defined. Although originally developed for notational systems, these typologies can also be adapted to describe systems in other formats, including body-based systems [7,26]. In line with the latter, we adopt the typology proposed by Widom & Schlimm [13] and extend the analysis of the type of representation not just for full bases, but for all main anchors.

Accordingly, anchor representation is *integrated* if just *one type* of sign represents both the numerical value of the main anchor and its multiplier. For instance, 200 is represented in the (ciphered) Greek notational system with a single sign of one type (as ‘σ’) and in the (cumulative) Roman system with accumulated signs of one type (as ‘CC’—each sign in the numeral is simultaneously part of the multiplier 2 and a symbol for base 10^2^). For a body-based example of integrated anchor representation, see 10, 20, 30 and 100 in the Pokot system above.

Anchor representation is *parsed* if *different types* of signs represent the main anchor and its multiplier, respectively. This involves using distinct number words in verbal systems (e.g. ‘two hundred’ in English) and distinct signs in notational systems (‘二百’ in Chinese). In body-based systems, a movement can represent the anchor and a hand shape the multiplier, as in the Pokot numerals for 40 and 50.

Finally, anchor representation is *positional* if only the multiplier is explicitly represented, whereas the value of the main anchor is indicated by position. In the Western notational system, for instance, only the positions of the numerals ‘0’ and ‘2’ in ‘200’ indicate that the numerical value is 200. In body-based systems, position can be implemented by assigning new powers to different hands (e.g. [3]), fingers [30], positions in space [31] and even other people [32].

## 3. The *BodyBase* database and the subset used here

The current study is based on a subsection of systems from the *BodyBase* database—an extensive database still under development that documents various aspects of, to date, 813 body-based numeration systems. The subsection used here consists of those 480 (59%) systems for which data on compositionality is available, that is, on whether or not the system employs any anchors and is accessible on the Open Science Framework platform [33].

The *BodyBase* database contains data that characterize properties of body-based numeration systems: conventionalized cultural practices of representing numbers using various body parts such as fingers, toes, points on the upper and lower limbs, head and torso. It considers applications for counting, tallying, montring and finger arithmetic systems, irrespective of whether a system was culturally developed and transmitted or whether it was designed relatively recently. However, we exclude systems whose descriptions do not explicate the initial counting sequence starting from 1, and describe counting in groups of, say, 10 only. Occasionally, a system’s structure is influenced by coexisting systems in other representational formats such as verbal representations. In such cases, for this paper, we focus exclusively on the body-based representations. We also exclude numeration systems in sign languages, owing to their distinct linguistic nature [4,34].

### (a) Data collection and categorization

We conducted an extensive literature review focussing on ethnographic, linguistic, cognitive and anthropological publications as well as videos that are openly available online. We mainly targeted digital resources, including archive.org, Google Books/Scholar, open-access databases and the online ethnographic archive eHRAF World Cultures (https://ehrafworldcultures.yale.edu/).

Each system or system variant is assigned a unique alphanumeric identifier (ID). Each entry is identified by the system’s name, if existent (e.g. Chisanbop), or the name of the cultural group using the system (e.g. Arusha Maasai). When groups use multiple systems, such as an old and a modern one, each receives a distinct ID. Glottocodes (https://glottolog.org/) are used to facilitate cross-referencing with other databases [35].

### (b) Variables of interest

For this study, we focus on the variables that characterize compositional anchors, specifically the anchor’s type, numerical value and representation (for formal definitions, see [22]).

*Types of anchors*:

(i) *main anchor*: the number whose numeral is most frequently and regularly used to compose non-atomic numerals; regular anchors reappear in at least half of the numerals corresponding to its value (e.g. a sign for 5 is regular if it occurs in at least three other numerals within a counting cycle);
(ii) *base*: a main anchor in a system that distinctly designates also at least the second power of that anchor;
(iii) *sub-base*: a regular anchor in a base system, where the base is a multiple of this number, and where shifts in the counting unit above the base are multiples of both the base and sub-base;
(iv) *sub-anchor*: equivalent to sub-base, but in systems without a base;
(v) *secondary anchor*: an anchor that is regular, but less frequent than the main anchor (co-exists with a main anchor); and
(vi) *sporadic anchor*: an atomic numeral only used irregularly to compose non-atomic numerals.

*Types of representations of main anchors (and their powers, where they exist*):

(i) *integrated*: the anchor and its multipliers are represented by only one type of sign;
(ii) *parsed*: the anchor and its multipliers are represented by different types of signs; and
(iii) *positional*: the anchor is represented by a position and its multipliers by a sign.

## 4. Compositional patterns in body-based numeration systems

**Figure 1.**
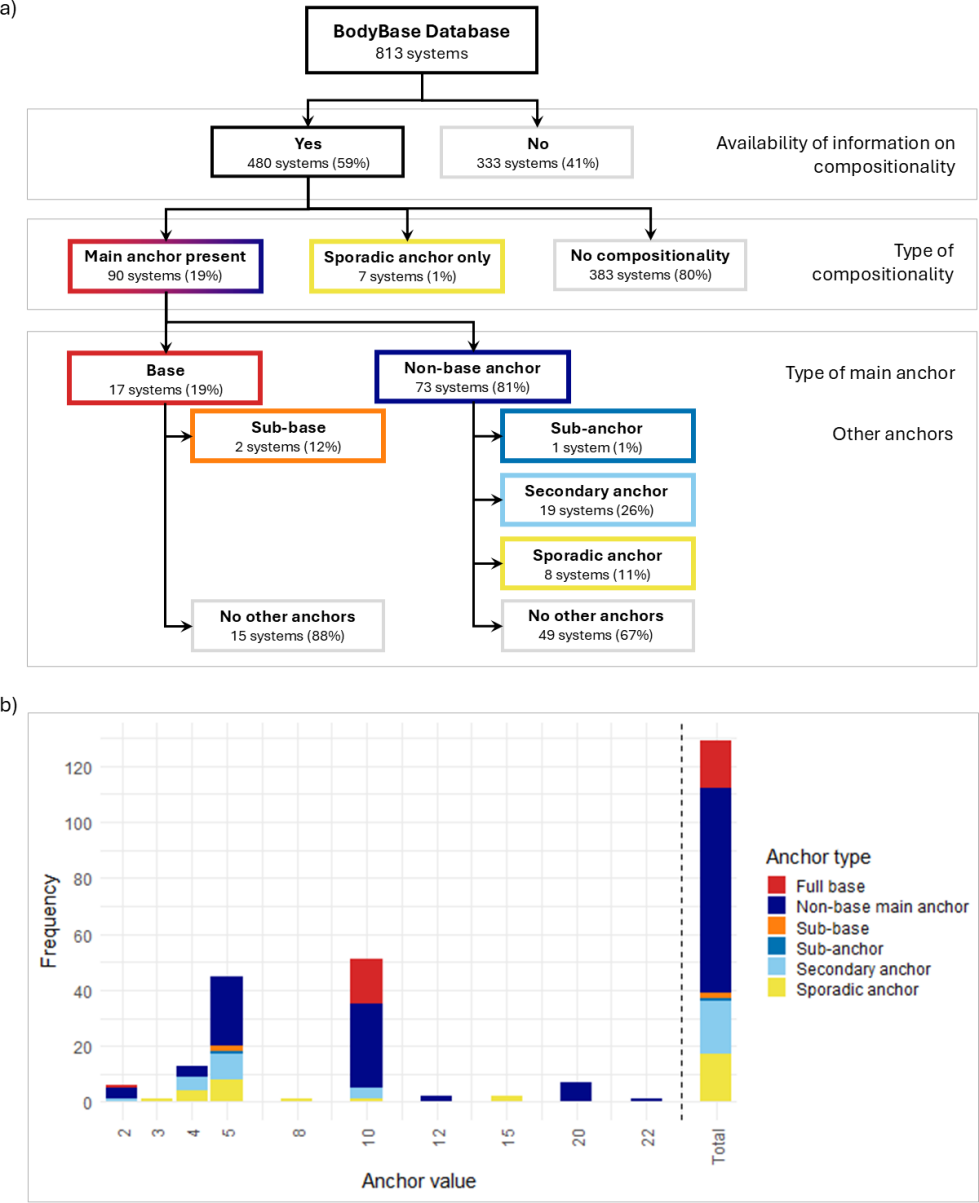
(a) Proportion of systems with compositional structure and distribution of different anchor types. (b) Frequency of values adopted by each anchor type.

As mentioned earlier, the 480 of the 813 systems in *BodyBase* (59%) containing data on compositionality constitute the dataset for this study (see figure 1a), irrespective of the systems’ intended purpose, origin or variant. In the following sections, we present what these data reveal about the distribution of anchor types and numerical values (§§4a,b) and about how various main anchors are represented (§4c).

### (a) Types of compositional anchors

Most (80%) of the systems for which information on compositionality is available lack any type of anchor. In 90 (19%) systems, a main anchor can be identified, yet only 17 of these qualify as proper base (less than 4% of all systems). Sub-bases and sub-anchors are even rarer, with just two systems having a sub-base and one having a sub-anchor. Secondary anchors appear in 19 systems (26% of those with a main anchor), but not in systems with a base. Sporadic anchors, finally, appear at two levels, either accompanying other anchors in a system with a non-base main anchor (eight instances = 11% of those systems), or as the sole anchor in otherwise unstructured systems (seven instances = 1% of all systems).

### (b) Numerical value of compositional anchors

Anchors across types adopt different values, but the observed variety is constrained (figure 1b). In our dataset, only 10 numbers serve as anchors, with few serving more than one type and none appearing across all types. Moreover, certain numbers are more likely to serve as particular types of anchors than others: while 3, 8 and 15 serve only as sporadic anchors, and 12, 20 and 22 only as non-base main anchors, 2, 4, 5 and 10 can assume multiple roles. Notably, 5 can function in nearly all types but base. This parallels notational [11] and verbal systems [21], where 5 is almost exclusively employed as a sub-base.

Main anchors (including bases) most frequently have a value of 10 (present in 46 systems, i.e. more than half of those with a main anchor), followed by 5 (in 25 systems) and 20 (in 7 systems), but can also take 2, 4, 12 and 22. Except for one instance,^2^ all base systems (*n* = 16) adopt 10 as the base, and all sub-bases and sub-anchors adopt 5. Secondary anchors, too, vary in the values they can take, but are restricted to 2, 4, 5 and 10. Sporadic anchors take six different values, and they typically appear in specific numerals and limited numerical ranges. Based on our data, 3 is exclusively used in the composition of the numeral for 6, while 8 serves as an anchor only in the numeral for 10. Similarly, 4 appears sporadically in numerals for 7 to 9, 5 in numerals below 10, 10 in numerals below 15 and 15 in the range from 16 to 19.

**Figure 2.**
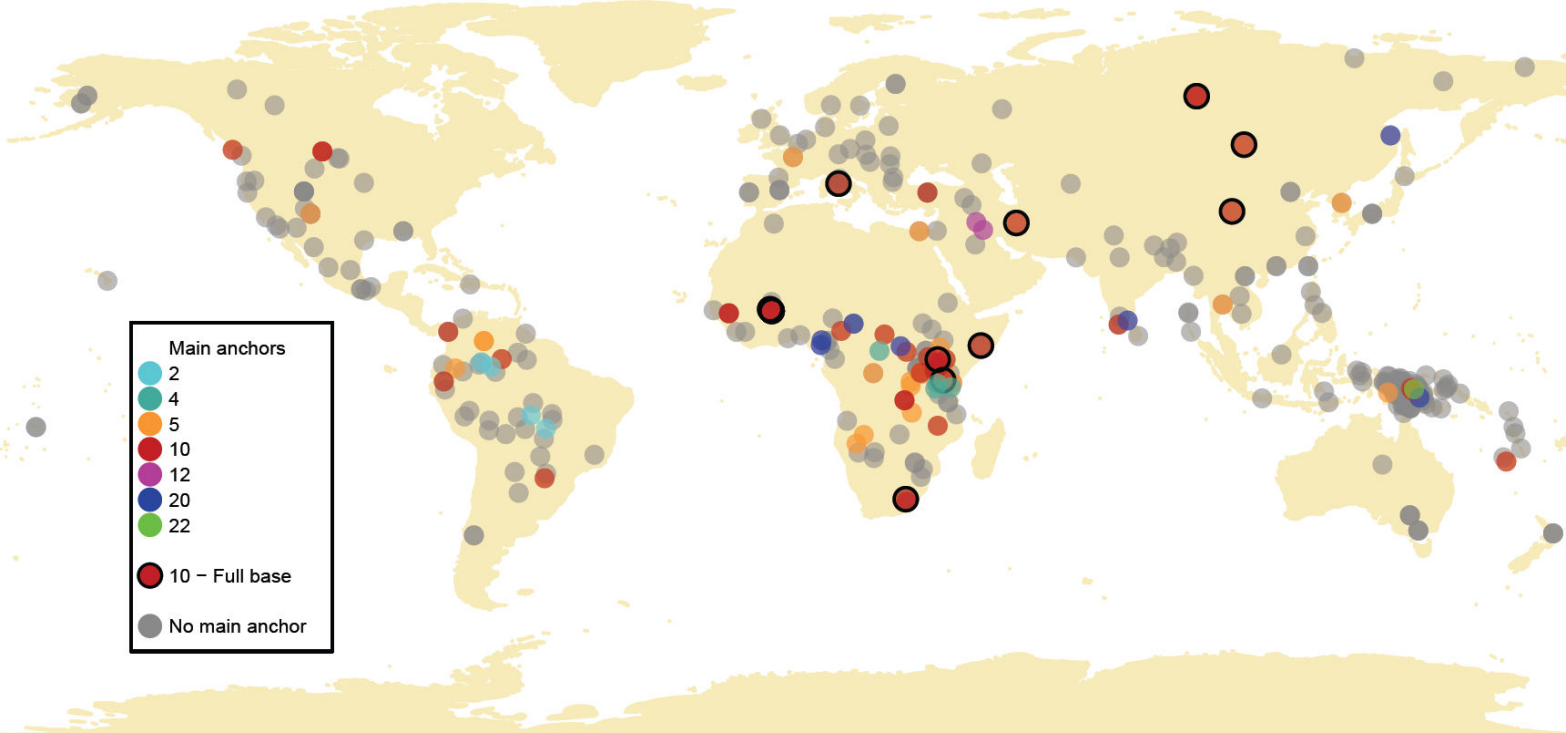
Global distribution of those systems in *BodyBase* for which data on compositionality are available (*n* = 455 plotted), grouped by main anchor value (colour-coded), including bases (circled in black). Locations are based on Glottolog coordinates. Note that for geographically widespread and/or highly mobile groups, locations are approximate estimates; and for those for which we lack coordinates, the nearest relative (either parent or child languoid) was used as approximate location. Not represented on the map are systems with unspecified locations and those used by groups that are too numerous to be accurately plotted (*n* = 25).

As shown in figure 2, some values of main anchors are only attested in specific geographical regions, with 2 appearing as the main anchor predominantly in systems in South America, 4 in Central and East Africa and 12 in the Middle East. By contrast, 5 and 10 are widespread and present on all continents except Australia. Regions also vary in terms of local diversity. For instance, while most systems in Oceania lack a main anchor (with a few notable exceptions), African systems are proportionally more diverse with regard to anchor values and their distribution across the region.

### (c) Representation of main anchors

The final property addressed here is the representation of main anchors, including bases. In most of our systems, the anchor is represented either in an integrated (*n* = 65) or positional (*n* = 14) manner, whereas purely parsed representation is extremely rare (*n* = 1). Ten systems combine two types of representation, namely integrated with either parsed or positional (see table 2).

**Table 2.** Frequency of different types of representations of the main anchor (including bases), grouped by its value.

value of main anchor	frequency of main anchor representation					totals (%)
	integrated	parsed	positional	integrated and parsed	integrated and positional	
2	4	—	1	—	—	5 (5.6)
4	4	—	—	—	—	4 (4.4)
5	22	—	3	—	—	25 (27.8)
10	28	—	8	4	6	46 (51.1)
12	2	—	—	—	—	2 (2.2)
20	4	1	2	—	—	7 (7.8)
22	1	—	—	—	—	1 (1.1)
totals (%)	65 (72.2)	1 (1.1)	14 (15.6)	4 (4.4)	6 (6.7)	90 (100)

#### (i) Integrated representations

Integrated representation is by far the most prevalent, and it can be achieved through various strategies. First, different units can be assigned to distinct body parts; for example, a finger segment can denote a value of 1, and a shift in counting unit can be marked by switching to counting whole fingers of another hand [36]. Second, the anchor can be represented by a static gesture, and multiples of that anchor can be expressed by repeating that gesture or implementing new ones (as in the Pokot system). Finally, systems can use dynamic expressions, such as clapping hands or joining fists to represent a number and repeating this gesture to denote its multiples. Interestingly, some instances of integrated representation preserve cumulative aspects of the numbers. For example, a fist that designates 5 still contains five bent fingers, and clapped hands contain 10 fingers. Conversely, the integrated representation of the anchor can also take the form of more ciphered representations (adhering less to a one-to-one correspondence with the number they represent).

#### (ii) Parsed representations

Parsed representations are particularly frequent in verbal systems but almost absent in body-based systems, despite being technically feasible (e.g. by harnessing two different gestures or hand shapes). In the five cases in which parsed representation does occur (either exclusively or in conjunction with an integrated representation), this is achieved through distinct movements that accompany a hand shape and thereby modify its numerical value. One of the most elaborate systems employing this strategy is found among the Arusha Maasai, where a variety of movements repurpose the symbols for units so as to assign them values in different powers; for instance, the representation of 60 resembles that of 6 repeated multiple times [37,38]. It is worth noting that such movements need not be consistently applied across the entire system and usually vary in different numerical ranges.

#### (iii) Positional representations

Although it is possible to represent the main anchor positionally through a sequential combination of signs (e.g. a gesture for 4 followed by a gesture for 2 to denote 42), this type of representation is rarely attested in our sample, where it is largely confined to merchant transactions [39]. A much more frequent strategy is to simply assign powers of the base to specific fingers, as in the Chinese base−10 [30,36] and the above-mentioned base−2 system [7]. In the former, the numerals for 1–9 are distributed across different parts of a finger’s segments, with higher power levels assigned to subsequent fingers. The binary system assigns just two values, 0 and 1, to bent and extended fingers, respectively, and represents composite numerals by combining consecutive fingers. Both systems transition to the second-hand based on spatial proximity, that is, by representing consecutive powers by the nearest finger on the next hand.

Positional representation can also be achieved by assigning one hand to represent the primary counting cycle while the other hand tracks multipliers of the anchor or its higher powers, reusing previously employed gestures. The distinction between left and right is crucial, as the hand shapes remain the same. Some systems use additional visual cues, such as positioning hands differently in space to represent varying ranges of numbers [31].

Finally, positional representation can be achieved by including additional people to be counted on. In such cases, one person tracks the primary counting cycle up to the anchor and another person keeps track of its multiples, using exactly the same gestures [40]. Additional people can also represent raising powers of the base. Each person then shows one numeral of each power, and the numerical meaning relies on the position of each individual in physical space (e.g. [32]).

## 5. Discussion

The current paper illustrates the compositionality of body-based numeration systems using data from *BodyBase*, focussing on anchor types and numerical values and on how main anchors are represented. Previous work by Bender & Beller [7] illustrated the range of compositionality in 18 systems, highlighting various strategies that shape systems' structures. Overmann [41] analysed 28 systems, identifying most as predominantly one-dimensional and lacking a clear main anchor. With an analysis based on 480 systems, we are able to provide a much more comprehensive view.

This view may not give the full picture either. For almost half of the 813 systems in *BodyBase* so far, information on compositionality was not available, and some regions (notably Australia) are under-represented. This is largely owing to a lack of detail in many sources and a research focus that is biased towards certain groups. Partly for the same reason, the categorization of individual numerals and entire systems is not always unequivocal. Often, gestures for numbers used in conjunction with verbal expressions or as a communal practice involving multiple individuals complicate their evaluation as stand-alone tools for quantification. Furthermore, not all of the anatomically shaped features are captured by existing typologies.

None of these limitations, however, compromise our main findings regarding the diversity and creativity in extending body-based numeration systems through the use of compositional anchors. We begin our discussion with general patterns, before comparing our systems with those in other representational formats.

### (a) General patterns

To begin with, most body-based numeration systems are not compositional; that is, they do not use anchors as part of their structure. This suggests that their main purpose may not have been to keep track of large quantities. Note, though, that non-compositional systems—particularly some body-counting systems in New Guinea (e.g. [42,43])—can still extend way beyond 10 and even 20 (the highest reaching up to 74, although 27 is the most common; see [44]). The variability in how numbers are represented in non-compositional systems is also extensive, encompassing differences in the starting hand or finger and the techniques used to represent consecutive numbers [41,45]. Among the compositional systems, there is much greater diversity than hitherto recognized, which corresponds to a view of numeration systems as cultural tools, attesting to human creativity in finding solutions to practical and cognitive challenges [19]. This diversity includes the types of anchors used, the numerical values they adopt and the way in which they are represented. Importantly, this diversity is not limitless, and some general patterns can be observed.

First, most systems with any type of anchor have (non-base) main anchors, and these also constitute the largest proportion of all anchors. In other words, those systems that have a compositional structure are generally structured in a *systematic* manner, which not only facilitates their cognitive handling but also opens them up for further extension if needed.

Second, 10 and 5 appear as anchors most frequently—the former mostly as either a base or non-base main anchor, the latter in various roles *except for* base. This suggests a preference for the 10 (full) fingers as the primary means for number representation in many, even if far from all, systems in our database.

Third, the representation of main anchors is prevailingly integrated or positional, yet realized through a variety of distinct methods. Different types of representations can even co-occur within the same system. It was previously suggested that certain format-specific aspects of body-based representations, such as the rules for switching between hands or gestural movements, have a limited influence on a system’s structure [9]. On the contrary, our data reveal that format-specific aspects can play an active role in composing larger numerals and hence affect how the system is structured. In parsed representations, in particular, movements added to a numeral symbol have a numerical value of their own, while in integrated representations, the movement is inseparable from the hand shape and has no independent numerical value. Additionally, positional representation can be achieved by either prioritizing the nearest fingers on the second hand or by closely mirroring the gesture from the first hand. Thus, the method of transitioning between hands becomes a key factor in how numbers are represented.

### (b) Across representational formats

As mentioned in §2, one long-term goal of our work is to compare body-based numeration systems with those in other representational formats (for how this can be supported by visualizing system structure in a joint format, see [10]). While detailed comparisons are not yet feasible, some general observations can be made with regard to similarities, differences and interactions across formats.

The overall pattern of compositionality in body-based systems shows some similarities to how numbers are represented in verbal and notational systems. Numeration systems across formats frequently use 10 and 20 as main anchors or bases and 5 as sub-bases [11,20,46,47]. As the human body comes with two hands and two feet, comprising five digits each, it has been suggested as the primary candidate for the conceptualization of numbers and a crucial building block of numeration systems [48]. Our findings suggest that body anatomy may indeed strongly influence the structure of at least the body-based numeration systems, even if not all systems take the collection of 10 whole fingers as their primary means for representing numbers. Additionally, rare anchors such as 3, 4, 8, 12 and 15, found in verbal systems [18], also appear in our sample, suggesting that body-based systems might be closely related to language or more general cultural influences.

We observed that body-based systems often rely on cumulative representation of anchors and their multipliers (e.g. clapping several times to denote multiples of 10), which is a common feature also in notational systems [11] but not in verbal systems. Furthermore, while positional representation of numbers is shared between notational and body-based systems, the latter employ a wider range of strategies to achieve this, though also being more limited in the number of positions available for representing numbers.

Body-based numeration systems, however, often display some level of irregularity and may incorporate multiple competing features, such as employing various representation types within the same system or using secondary and sporadic anchors. This makes them more akin to how numbers are represented in verbal systems than in notational systems. Additionally, the frequent use of non-base main anchors further distinguishes body-based systems from notational systems.

Parsed representations, while commonly found in both verbal and notational systems, are exceedingly rare in body-based numeration systems. Sign languages, by contrast, make use of this type of anchor representation in their numeration systems, labelled as numeral incorporation [49]. Thus, the rarity of parsed representations in body-based systems cannot merely be explained by the format of representation.

These similarities and differences illustrate the complex relationships between the structural properties of numeration systems and their representational formats (see also [46]). The fact that formats differ in complementary manners may also be one reason why number representations sometimes combine elements from systems implemented in different formats.

Verbal cues are often used alongside gestures to help clarify or supplement their numerical value. In many cases, verbal counting simply complements the physical gestures, providing an additional layer of information that helps to disambiguate the intended number [37,40]. In other cases, this interaction can create hybrid systems with complex properties that unfold across multiple formats simultaneously (e.g. [50]). Also, in other cases still, body-based and verbal systems are so closely intertwined that context information is indispensable for understanding their structural architecture.

This integration of verbal cues with gestures highlights the cognitive flexibility afforded by multiformat or ‘multimodal’ communication [51]. Different formats might serve distinct purposes, such as using speech in the dark or gestures in silence. They probably evolved to meet various practical needs and hence may vary depending on the contexts in which they are used.

## 6. Conclusion

Body-based numeration systems need not be restricted to counting to 10 on one’s fingers. Around the globe, people using such systems have found ways to extend them, by employing compositional anchors to construct composite numerals, and they have done so by creatively harnessing various format-specific features and strategies. Compositional anchors vary substantially across cultures in terms of their type, numerical value, and representation. Distinguishing more finely between different types of compositional elements is especially important for body-based numeration systems, as it allows for a more detailed and precise description of their diversity. Upon such closer and thorough examination, body-based numeration systems turn out to be a surprisingly complex, intriguing and challenging topic of study that pushes the boundaries of our understanding of how human cognition and cultural practices interact, and of the diverse ways in which communities conceptualize and represent numbers.

## Data Availability

The dataset and R code supporting this article have been uploaded to OSF: [33].

## Footnotes

^1^
When used without quotation marks, Western (Indo-Arabic) numerals refer to numbers.

^2^
An exceptional case in our database is the one instance of a proper base-2 system, which is a hypothetical system; described by Bender & Beller [7] and on a Wikipedia page of its own (https://en.wikipedia.org/wiki/Finger_binary). In naturally occurring systems, base 2 is very rare across representational formats.
